# Insight into GEBR-32a: Chiral Resolution, Absolute Configuration and Enantiopreference in PDE4D Inhibition

**DOI:** 10.3390/molecules25040935

**Published:** 2020-02-19

**Authors:** Valeria Cavalloro, Katia Russo, Francesca Vasile, Luca Pignataro, Archimede Torretta, Stefano Donini, Marta S. Semrau, Paola Storici, Daniela Rossi, Federica Rapetti, Chiara Brullo, Emilio Parisini, Olga Bruno, Simona Collina

**Affiliations:** 1 Department of Drug Sciences, University of Pavia, 27100 Pavia, Italy; valeria.cavalloro01@universitadipavia.it (V.C.); katia.russo01@universitadipavia.it (K.R.); daniela.rossi@unipv.it (D.R.); 2 Department of Earth and Environmental Sciences, University of Pavia, 27100 Pavia, Italy; 3 Università degli Studi di Milano, Dipartimento di Chimica, via C. Golgi 19, 20133 Milano, Italy; luca.pignataro@unimi.it; 4 Center for Nano Science and Technology @PoliMi, Istituto Italiano di Tecnologia, Via Pascoli 70/3, 20133 Milano, Italy; archimede.torretta@iit.it (A.T.); stefano.donini@iit.it (S.D.); emilio.parisini@iit.it (E.P.); 5 Elettra-Sincrotrone Trieste S.C.p.A., SS 14-km 163.5 in AREA Science Park, 34149 Trieste, Italy; marta.semrau@elettra.eu (M.S.S.); paola.storici@elettra.eu (P.S.); 6 Department of Pharmacy, Section of Medicinal Chemistry, School of Medical and Pharmaceutical Sciences, University of Genoa, 16132 Genoa, Italy; federica.rapetti@edu.unige.it (F.R.); brullo@difar.unige.it (C.B.); obruno@unige.it (O.B.); 7 Latvian Institute of Organic Synthesis, Aizkraukles 21, LV-1006 Riga, Latvia

**Keywords:** HPLC chiral resolution, NMR absolute configuration assignment, PDE4 inhibition

## Abstract

Alzheimer’s disease is the most common type of dementia, affecting millions of people worldwide. One of its main consequences is memory loss, which is related to downstream effectors of cyclic adenosine monophosphate (cAMP). A well-established strategy to avoid cAMP degradation is the inhibition of phosphodiesterase (PDE). In recent years, GEBR-32a has been shown to possess selective inhibitory properties against PDE type 4 family members, resulting in an improvement in spatial memory processes without the typical side effects that are usually correlated with this mechanism of action. In this work, we performed the HPLC chiral resolution and absolute configuration assignment of GEBR-32a. We developed an efficient analytical and semipreparative chromatographic method exploiting an amylose-based stationary phase, we studied the chiroptical properties of both enantiomers and we assigned their absolute configuration by ^1^H-NMR (nuclear magnetic resonance). Lastly, we measured the IC_50_ values of both enantiomers against both the PDE4D catalytic domain and the long PDE4D3 isoform. Results strongly support the notion that GEBR-32a inhibits the PDE4D enzyme by interacting with both the catalytic pocket and the regulatory domains.

## 1. Introduction

**Figure 1:**
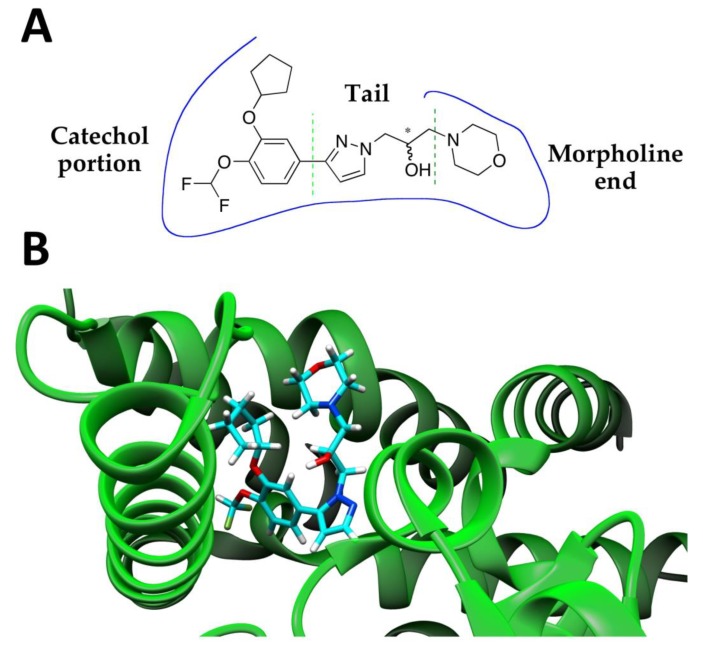
(**A**) Chemical structure of GEBR-32a and (**B**) crystal structure of *S*-GEBR-32a in complex with the phosphodiesterase type 4 (PDE4D) catalytic domain (PDB 6FDC).

Nowadays, more than 30 million people live with dementia worldwide, and their number is estimated to grow to 65.7 million in 2030 and 115.4 million in 2050 [1]. As a result, dementia will probably reach third place in terms of total healthcare costs, after cancer and cardiovascular diseases [2]. Among all, Alzheimer’s disease (AD) represents the most common form of dementia and it can be considered a challenge under both a medical and an economical point of view [3]. AD is a polygenetic neurodegenerative disorder characterized by neocortical atrophy that causes the loss of synapses and neurons [4]. One of its main consequences is memory loss, which is strictly related to downstream effectors of cyclic adenosine monophosphate (cAMP) [5]. Phosphodiesterase (PDE) inhibition has long been considered a promising strategy to avoid the hydrolysis of cAMP and, therefore, to treat memory deficits [6]. Particularly, inhibitors of the PDE type 4 (PDE4) family have been identified as effective pro-cognitive drugs [7,8], and different drug discovery programs aim at developing an effective PDE4 inhibitor to treat memory disorders. However, PDE4 inhibitors have been associated with severe adverse effects, mainly emesis. Recently, it has been demonstrated that PDE4D is more relevant to cognition enhancement than other PDE4 isoforms [9]. Thus, the quest for potent and selective PDE4D inhibitors could open new avenues for memory performance enhancement. By combining X-ray crystallography, molecular dynamic simulations and enzymatic assays, members of our research team have recently provided a structural and functional characterization of several PDE4D inhibitors from the well-established GEBR library [10]. Among them, GEBR-32a (Figure 1) holds particularly good promises. Indeed, this PDE4D selective inhibitor is characterized by a good toxicological and pharmacokinetic profile, and it is able to improve spatial memory processes without undesired emetic-like side effects [7]. From a structural standpoint, we have shown previously that the catechol portion of GEBR-32a binds the catalytic domain, while the tail of the molecule extends into the S pocket, where it makes water-mediated contacts with the surrounding residues, and develops toward the entrance of the active site (PDB 6FDC) [10]. Hence, when bound to the catalytic domain, the protruding tail of GEBR-32a is expected to interact with the regulatory domains of the enzyme (UCR1/UCR2 or CR3), which cap the catalytic pocket. Indeed, as the IC_50_ value measured against the PDE4D catalytic portion alone is higher than the IC_50_ value against the full-length PDE4D3 isoform, we hypothesized that the tail of GEBR-32a contributes to its inhibitory mechanism [10]. Since the tail portion of GEBR-32a is characterized by the presence of a stereogenic center, the absolute configuration may also play a role in the interaction with the enzyme. Interestingly, despite the fact that the structure of the PDE4D catalytic domain in complex with GEBR-32a has been determined using the GEBR-32a racemic mixture, the crystallographic analysis provided evidence of a preferential interaction of *S*-enantiomer with the enzyme (Figure 1B) (PDB 6FDC). This observation suggested that (*S*)-GEBR-32a may be the eutomer and provided the rational for further careful investigation on the resolved enantiomers.

In line with these considerations, herein we report on the chiral resolution of racemic GEBR-32a, on the absolute configuration (AC) assignment and on the comparison of the inhibitory potency of the two enantiomers against both the PDE4D catalytic domain and the full-length PDE4D3 isoform.

## 2. Results and Discussion

### 2.1. Chiral Chromatographic Resolution

**Figure 2:**
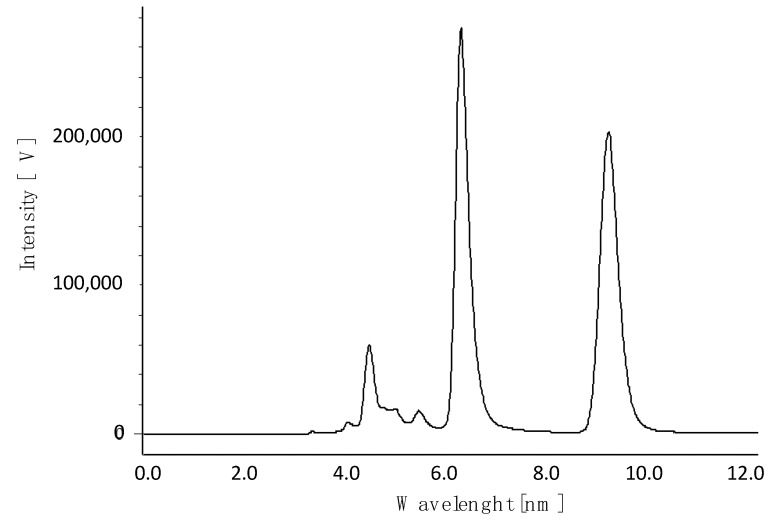
Analytical profile of racemic GEBR-32a: Regispack IA (150 mm × 4.6 mm, 5 µm) and 100% IPA plus 0.1% DEA at a flow rate of 0.5 mL/min (λ = 280 nm). N/A: not applicable due to the low poor resolution.

Considering that both enantiomers are needed for biological testing, we synthesized racemic GEBR-32a first (according to the previously reported procedure, Scheme SI) [7] and solved the enantiomers by chiral liquid chromatography, a time- and cost-effective approach for enantiomer resolution at the discovery stage, commonly used both in Academia and the pharmaceutical industry [7,11]. To find easily scalable chiral resolution analytical methods, our standard approach to the analysis and separation of new chiral compounds consisted in the screening of polysaccharide-based chiral stationary phases (CSPs) immobilized on silica gel chiral columns characterized by wide solvent compatibility, versatility and robustness [11,12,13,14,15,16,17,18]. First, we tested a Regispack IA column containing a dimethylphenyl-derivative of amylose. An initial mobile phase consisting of *n*-hexane (HEX)/isopropyl alcohol (IPA)/diethylamine (DEA) (90:10:0.1) was used in order to assess the ability of the chiral stationary phase (CSP) to resolve the GEBR-32a racemic mixture. Giving the encouraging resolution thus obtained, a complete screening protocol (Table 1) in polar organic and reversed-phase mode was applied under isocratic elution conditions. The best results in terms of peak resolution, peak shape and enantioselectivity were achieved by eluting with isopropyl alcohol supplemented with 0.1% DEA at a 0.5 mL/min flow rate. These chromatographic conditions allowed for very short retention times and analysis time, adequate for scaling up (Figure 2).

In Table 1, the results expressed as retention (k), selectivity (α) and resolution (Rs) factors are reported.

This method was transferred to a (semi)preparative scale using the Chiralpak IA, ID of 10 mm, characterized by the same CSP of the analytical column used. The good solubility of the racemate in the mobile phase allowed the fast separation of the enantiomers. About 70 mg of racemic GEBR-32a were processed in seven runs, and both enantiomers obtained with high enantiomeric excess (*ee*) and in suitable amount for further studies (more than 30 mg for each enantiomer). As evidenced by the analytical control of the collected fractions (Figure S1), the first eluted enantiomer has an *ee* of 99.5%, and the second one of 99.9%. The recovery is reported in Table 2 and the (semi)preparative chromatographic profile in the supplementary material (Figure S1).

**Figure 3:**
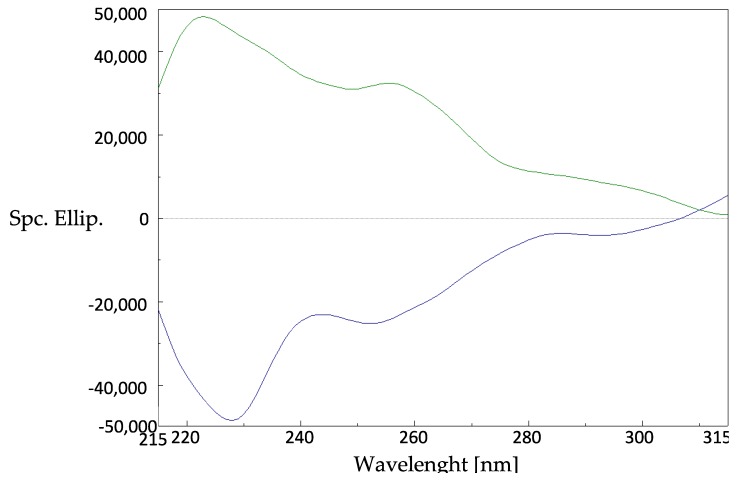
Circular dichroism spectra of (+)-GEBR-32a (green line) and (−)-GEBR-32a (blue line). Analysis are performed in methanol at *c*: 1.37 × 10^−4^ M.

Both enantiomers have been characterized by nuclear magnetic resonance (NMR) (^1^H-NMR and ^13^C-NMR) by measuring the specific rotations (${\left[\alpha \right]}_{D}^{20}$) and recording electronic circular dichroism spectra (Table 2 and Figure 3).

The electronic circular dichroism (ECD) spectra of GEBR-32a showed two cotton effects (CEs) between 215 and 300 nm corresponding to the characteristic 1La and 1Lb transitions of the benzene chromophore. The first eluted enantiomer, (–)-GEBR-32a, showed negative cotton effects, and, as expected, the second eluted enantiomer exhibited an identical ECD profile but of opposite sign (Figure 3).

### 2.2. Assignment of the Absolute Configuration (AC)

The absolute configuration (AC) assignment of enantiomeric GEBR-32a was performed by nuclear magnetic resonance (NMR) spectroscopy. The strategy is based on the derivatization of the substrate with unknown configuration using both the enantiomers of a chiral reagent, leading to two diastereoisomeric derivatives. The assignment of the AC is obtained from the difference in chemical shifts observed in the ^1^H-NMR spectra of the two species [19].

**Figure 4:**
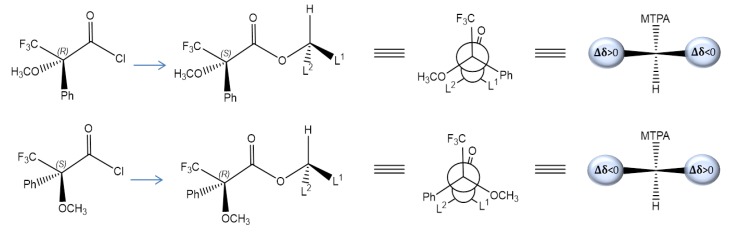
Generic (*S*)- and (*R*)-methoxy-α-trifluoromethyl-α-phenylacetic acid (MTPA) esters in their assumed preferred conformation, with the typical signs of Δδ = δ*_S_* − δ*_R_*.

**Scheme 1:**
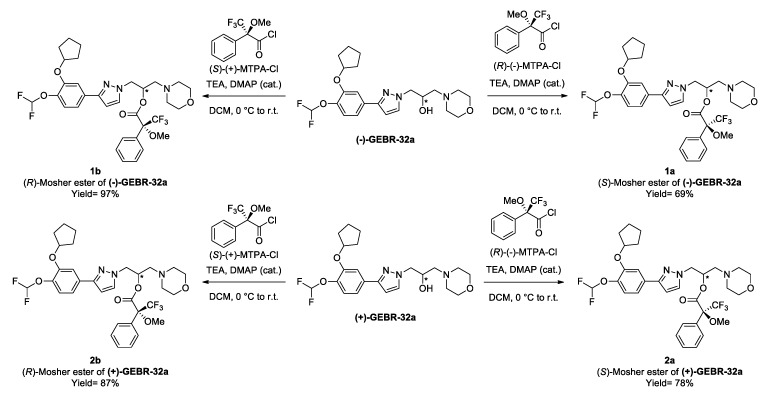
Synthesis of the four possible diastereosomeric forms of GEBR-32a Mosher’s esters. DCM: dichloromethane, DMAP: dimethylaminopyridine, MTPA: methoxy-α-trifluoromethyl-α-phenylacetic acid.

The presence of a hydroxy group directly connected to the stereocenter of GEBR-32a makes this molecule a suitable candidate for being derivatized by reaction with methoxy-α-trifluoromethyl-α-phenylacetic acid chloride (MTPA-Cl), also known as Mosher’s reagent and usually employed for the AC assignment of secondary alcohols and amines [20]. This methodology is well-established and has been exploited to assign the absolute configuration of a large number of different compounds [21]. Its rationale is based on the assumption that the diastereoisomeric esters obtained by derivatization adopt a *s-cis* conformation with the C=O and the CF_3_ group laying in the same plane [20,22,23]. In this conformation, the OMe and the Ph substituent of the MTPA moiety perturbate the signals of L^1^ and L^2^ groups belonging to the alkoxy residue (Figure 4): the aromatic ring will cause a high field shift of the substituent sitting on its side, while the substituent on the OMe side will remain unaffected or undergo an opposite change of δ. In the (*S*)-MTPA ester, the L^1^ group is close to the phenyl ring and so it resonates at higher field than in the (*R*)-MTPA derivative. At the same time, the chemical shift of L^2^ is larger in the (*S*)-MTPA ester (in which this residue is closed to the OMe moiety) than in the (*R*)-MTPA ester (in which it is close to the Ph ring). Thus, the chemical shift differences of all protons of the (*S*)- and (*R*)-MTPA derivatives are calculated, and, considering the projections shown in Figure 4, the stereocenter substituents are assigned as L^1^ and L^2^ depending on the sign of Δδ (defined as δ*_S_* − δ*_R_*). Following this general procedure, we derivatized the two enantiomeric alcohols as esters of both (*S*)-MTPA and (*R*)-MTPA (Scheme 1). To obtain the diastereomeric esters, small amounts of both (+) and (–)-GEBR-32a were reacted in parallel with (*R*) and (*S*)-α-methoxy-α-trifluoromethylphenylacetyl chloride (MTPA-Cl) [24]. The reactions were performed in dichloromethane (DCM) in the presence of triethylamine (4 equiv.) and of a catalytic amount of 4-dimethylaminopyridine (DMAP). It should be noted that—as illustrated in Figure 4—upon ester formation, the stereochemical descriptor of the MTPA-Cl stereocenter changes, because the COCl group has a CIP priority different from COOR.

Next, ^1^H-NMR experiments were performed on both the nonderivatized and the derivatized alcohols. The Δδ values of the protons involved in the interaction with MTPA are reported in Table 3.

The absolute configurations of the Mosher’s esters were assigned based on the Δδ signs, as described above. Considering that the absolute configurations and the stereochemical descriptors of GEBR-32a did not change upon derivatization, these results lead to conclude that the first eluted enantiomer is (*R*)-(–)-GEBR-32a and the second is (*S*)-(+)-GEBR-32a.

**Figure 5:**
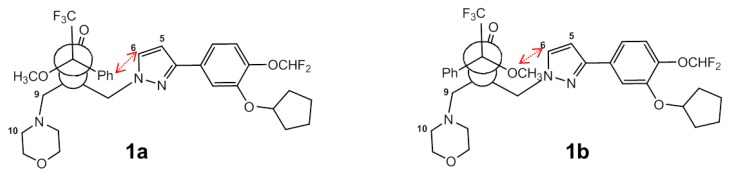
The most interesting NOE contacts observed for compounds Mosher’s esters **1a** and **1b.**

As already stated above, this assignment can be considered to be reliable only if the Mosher’s esters’ conformations in solution are actually those shown in Figure 4. In order to confirm this, we performed 2D-NOESY experiments, which can provide information on the relative position of their structural elements [25,26]. For compound **1a** ((*S*)-MTPA ester of (*R*)-GEBR-32a), we observed two important NOE contacts between the Ph ring of MTPA with protons 5 and 6 of GEBR-32a (Figure S2) and the OMe group of MTPA with proton 9 of GEBR-32a (see Scheme in Table 3 for atom numbering). On the contrary, in the NOESY spectrum of compound **1b** ((*R*)-MTPA ester of (*R*)-GEBR-32a), the OMe group of MTPA shows cross peaks with protons 5 and 6 of (*R*)-GEBR-32a (Figure S3), while the Ph ring of MTPA has interactions with protons 10 and 11. In Figure 5, the most significant NOE contacts are shown for compounds **1a** and **1b**. The NOESY spectra of the Mosher’s esters of (*S*)-GEBR-32a featured the following interactions: (a) for compound **2a** ((*S*)-MTPA ester of (*S*)-GEBR-32a), NOE contacts between the OMe group of MTPA and protons 5 and 6 of (*S*)-GEBR-32a (Figures S4 and S6), and (b) for compound **2b** ((*R*)-MTPA ester of (*S*)-GEBR-32a), NOE contacts between the Ph group of MTPA and proton 5 and 6 (*S*)-GEBR-32a (Figures S5 and S6).

Therefore, overall, the NOE data collected for these compounds are compatible with the assumed ester *s-cis* conformation with the C=O and CF_3_ groups eclipsed (Figure 4), suggesting that the latter is effectively populated in the solution.

### 2.3. Enzymatic Activity

**Figure 6:**
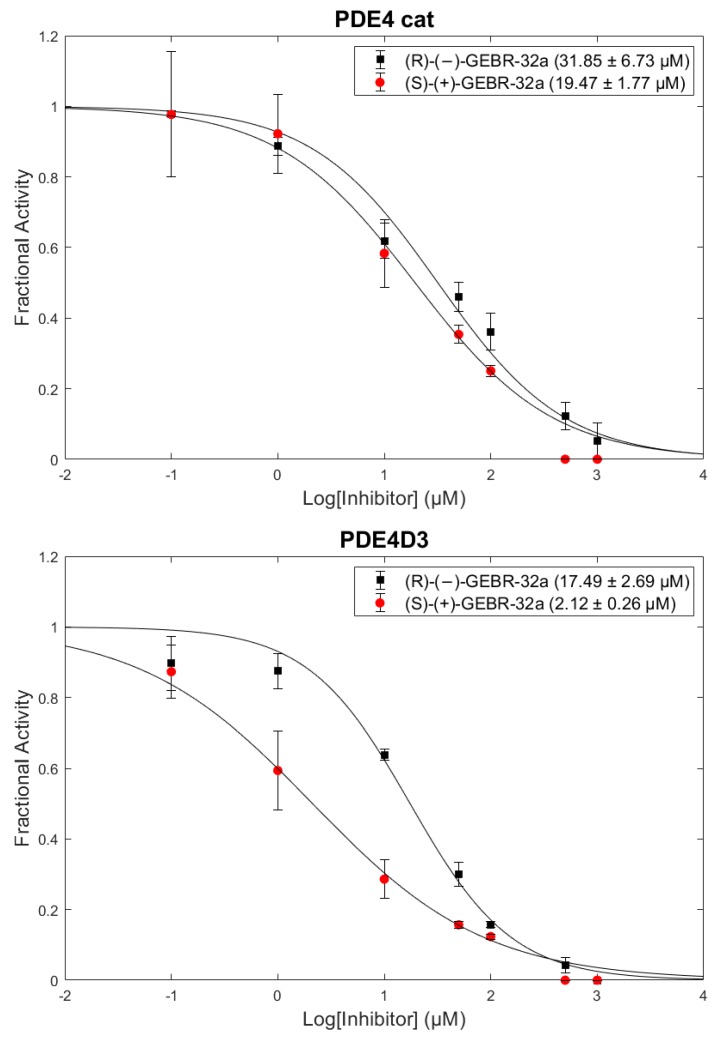
IC_50_ curves for the two GEBR-32a enantiomers relative to the catalytic domain only (**A**) and against the long PDE4D3 isoform (**B**). The experimental conditions are reported in the Materials and Methods section. The reported data are the mean values of three replicates ± SD (standard deviation).

With the enantiomers of GEBR-32a in hand, we evaluated their inhibitory activity on both the catalytic domain alone and the full-length enzyme. Results are shown in Table 4 and in Figure 6.

Results clearly show that inhibitory activity of GEBR-32a is significantly influenced by the configuration of the stereogenic center that is present in the tail portion. In fact, the (*S*)-configured enantiomer is approximately seven times more active than the (*R*)-configured one when measured against the full-length protein, whereas this fold-difference reduces to less than two when measured against the catalytic domain. This data confirms our hypothesis that the tail portion of GEBR-32a contributes significantly to the inhibition of the full-length PDE4D enzyme. In addition, enzymatic assays clearly indicate that (*S*)-GEBR 32a is the eutomer, in accordance with the crystallographic analysis, which evidenced a preferential interaction of *S*-enantiomer with the enzyme.

## 3. Materials and Methods

### 3.1. General

HPLC grade-solvents were supplied by Honeywell (Seelze, Germany), while analytical grade-solvents by PanReac (Darmstadt, Germany). The evaporation procedures were performed under reduced pressure using a Heidolph Laborota 4000 instrument (Heidolph Instruments GmbH & Co., Schwabach, Germany).

Analytical thin-layer chromatography (TLC) was carried out on silica gel precoated glass-backed plates (Fluka Kieselgel 60 F254, Merck, Darmstadt, Germany). The detection was conducted with UV light (λ = 254 nm).

Flash chromatography was performed with silica gel 60 (particle size 230–400 mesh) purchased from Nova Chimica (Cinisello Balsamo, Italy).

Deuterated solvent and all the reactants were purchased from Sigma Aldrich (Milan, Italy).

### 3.2. Chromatographic Resolution

Chromatographic resolution was carried out at room temperature on a Jasco (Tokyo, Japan) system consisting of a PU-1580 pump and MD-1510 photo diode array (PDA) detector.

Chromatogram acquisitions and elaborations were performed using the ChromNAV software (Tokyo, Japan). The resolution factor was directly given by the software, while selectivity (α) was calculated using Equation (1):

(1) $$\alpha =\frac{k_{2}}{k_{1}}$$

where *k*_1_ and *k*_2_ are the retention factors of the two enantiomers calculated as the ratio between the retention time (*t_r_*) and the solvent front (*t*_0_) in (Equation (2)):

(2) $$k=\frac{t_{r}-t_{0}}{t_{0}}$$

The best enantiomeric resolution was achieved exploiting a Regispack IA (250 mm × 4.6 mm, 5 μm) column from Daicel Chemical Industries (Tokyo, Japan), a chiral stationary phase composed by amylose tris(3,5-dichlorophenylcarbamate). The elution was performed in an isocratic mode using 100% IPA + 0.1% DEA at a flow rate of 0.5 mL/min (injection volume = 10 μL).

A Hamilton (Reno, NV, USA) syringe (syringe volume: 2.5 mL; loop: 2 mL) was employed for (semi)-preparative chiral resolutions. In detail, the scale-up of the analytical conditions allowed us to set up the following method: Regispack IA (1 cm × 25 cm, 5 μm) as column and flow rate of 2 mL/min injection volume of 1 mL (concentration: 10 mg/mL).

### 3.3. Specific Rotation

The optical rotatory power was recorded using a Jasco photoelectric polarimeter DIP 1000 (Tokyo, Japan). The analysis was conducted using a 0.5 dm cell, methanol as solvent and a sodium lamp (λ = 589 nm).

### 3.4. Circular Dichroism

Electronic circular dichroism (ECD) spectra were recorded on a Jasco J-1500 circular dichroism spectrophotometer (Tokyo, Japan) from 315 to 215 nm. The methanolic solutions of (–)-GEBR-32a (*c*: 1.09 × 10^−4^ M) and (+)-GEBR-32a (c: 1.37 × 10^−4^ M) were analyzed in a nitrogen atmosphere with an optical pathway of 1 cm. ECD spectra were scanned at 200 nm/min with a spectral band width of 1 nm and a data resolution of 1 nm. For each measurement, 10 scans were taken and averaged, considering both enantiomers. ECD spectra of the solvent in the same experimental conditions were subtracted. Data are reported in Δε versus λ (nm) from knowledge of the cell pathlength and solution concentration.

### 3.5. Absolute Configuration Assignment

Under nitrogen, (*R* or *S*)-MTPA-Cl (7.1 mg, 1 eq) was added to an ice-chilled solution of (+)- or (–)-GEBR-32a, TEA (6.6 mg, 4 eq) and DMAP (cat) in dry DCM (0.2 mL). After 15 min, the ice bath was removed, and the mixture was stirred at r.t. (30 °C) overnight. The volatiles were removed at rotavapor and the products purified by flash chromatography (eluent: 97:3 DCM/EtOAc).

### 3.6. NMR Analysis

NMR experiments were performed at 298 K on a Bruker Avance III 400 MHz spectrometer (Milan, Italy). The NMR experiments were carried out in 500 μL of CDCl_3_. All proton and carbon chemical shifts were assigned unambiguously using bi-dimensional experiments (COSY, NOESY and HSQC), and the assignments are reported in Table S1. Edited ^1^H-^13^C HSQC experiments were performed to confirm and follow the resonances of carbons. Phase sensitive 2D-NOESY experiments with gradient pulses in mixing time were performed with a mixing time of 700 ms in order to observe homonuclear correlation via dipolar coupling.

### 3.7. Expression and Purification of PDE4D Catalytic Domain and PDE4D3

The PDE4D catalytic domain and the PDE4D3 isoform were expressed and purified, as described previously [10]. Briefly, the PDE4D catalytic domain (residues 244–578) featuring a C-terminal 6His-tag was cloned into a pET-3a vector (Merck Millipore, Darmstadt, Germany) and transformed into *Escherichia coli* BL21(DE3) pLysS cells (Thermo Fisher Scientific, Waltham MA, USA). Transformed cells were cultured at 37 °C in LB broth supplemented with 50 mg/L ampicillin until OD600 = 0.6. Protein expression was carried out overnight at 25 °C after induction with 0.5 mM isopropyl 1-thio-β-D galactopyranoside (IPTG). Cells were harvested by centrifugation and resuspended in 20 mM Tris-HCl pH 7.5 and 150 mM NaCl. After sonication, the soluble fraction was first purified by affinity chromatography using a preequilibrated Ni-NTA (Qiagen, Hilden, Germany) column. Elution of the His-tagged protein was carried out using the same buffer supplemented with 400 mM imidazole. The eluted sample was further purified by size-exclusion chromatography using a Sephacryl 100 HR HiPrep 26/60 column (GE Healthcare, Chicago, IL, USA) and by anion exchange chromatography using a HiPrep Q HP 16/10 column (GE Healthcare, Chicago, IL, USA). The final protein sample was dialyzed against 20 mM Tris-HCl pH 7.5 and 150 mM NaCl and its purity assessed by SDS-PAGE.

The codon-optimized gene-encoding human PDE4D3 was purchased from GenScript (Piscataway, NJ, USA). The construct, featuring a C-terminal 6His-tag and cloned into the pFastBac dual vector, was designed to include the PKA phosphomimetic Ser54Asp mutation and the Ser579Ala mutation, the latter preventing a known inactivating phosphorylation [27]. The bacmid was generated by transposition in *E. coli* DH10EMBacY (strain kindly provided by I. Berger, University of Bristol, Bristol, UK) [28]. High-titer recombinant baculovirus was obtained by transfecting Sf9 cells grown in suspension at a density of 0.8 × 10^6^ cell/mL with PEI MAX (Polysciences Europe GmbH, Hirschberg, Germany). The protein was expressed in Sf9 cells (1.5 × 10^6^ cells/mL) for 72 h at 27 °C. Cells were harvested by centrifugation and resuspended in 50 mMHepes pH 7.5, 500 mM NaCl, 10 mM MgCl_2_, 10% glycerol, 5 mM imidazole, 10µg/mL DNaseI, 1 mM TCEP and a protease inhibitor cocktail (Roche, Mannheim, Germany). After mild cell disruption by the Avestin homogenizer, the lysate was incubated for 5 min with benzonase nuclease (Merck Millipore, Darmstadt, Germany) and then clarified by centrifugation. The supernatant was loaded on Ni-NTA resin (Qiagen, Hilden, Germany) pre-equilibrated in 50 mM Hepes pH 7.5, 150 mM NaCl, 10% glycerol, 5 mM imidazole and 1 mM TCEP and incubated for 1 h. After extensive washing with wash buffer containing 20 mM imidazole, the protein was eluted with the 50 mM Hepes pH 7.5, 150 mM NaCl, 10% glycerol, 400 mM imidazole and 1 mM TCEP. The protein was later diluted in 100 mM Hepes pH 7.5, 10% glycerol and 1 mM DTT and immediately purified on HiTrap HP Q column (GE Healthcare, Chicago, IL, USA) over linear gradient with buffer containing 1 M NaCl. The final purity of the sample was assessed by SDS-PAGE.

### 3.8. PDE4 Activity and Inhibition Assays

To measure enzyme activity, we used a coupled assay in which the adenosine monophosphate (AMP) generated from the hydrolysis of cAMP was detected by a NADH-coupled enzyme system [29]. The assay was adapted to support a high-throughput format in 96 transparent polystyrene plates from Greiner Bio (Kremsmünster, Austria). The 250 μL reaction mixture consisted of 100 mM Hepes pH 7.5, 10 mM MgCl_2_, 10 mM KCl, 0.3 mM ATP, 0.6 mM PEP, 0.9 unit of pyruvate kinase, 0.90 unit of lactate dehydrogenase, 0.8 unit of myokinase and the PDE4 enzyme at an estimated final concentration of 10 nM. A preincubation step was carried out at 25 °C for 20 min. Then, the reaction was started by adding cAMP at a final concentration of 0.04 mM. The oxidation of NADH to NAD+ was monitored over time at 340 nm (NADHε_340_ = 6220 M^−1^ cm^−1^) in a Spark10M instrument (Tecan, Männedorf, Switzerland). One unit (U) is defined as the amount of enzyme required to hydrolyze 1 μmol of cAMP to 5′-AMP per minute, and specific activity was expressed as units per milligram of enzyme. Inhibition assays using the two enantiomers of GEBR-32a were done following the same protocol and by varying the compound concentrations from 0.1 µM to 1 mM. Each enantiomer was dissolved in DMSO and diluted in the assay buffer to a final concentration of 1%. Finally, the IC_50_ values of both enantiomers relative to the PDE4 catalytic domain and to the PDE4D3 isoform were determined by plotting the enzyme fractional activity against the logarithm of compound concentration. Curve fitting was done with a dose−response curve from OriginLab (OriginLab Corporation, Northampton MA, USA) using Equation (3):

(3) y=min+max−min1+10^(logIC50−x)

where y is the fractional activity of the enzyme in the presence of the inhibitor at concentration [I], max is the maximum value of y observed at [I] = 0 and min is the minimum limiting value of y at a higher inhibitor concentration. All data points were collected in triplicate.

## 4. Conclusions

GEBR-32a is a promising phosphodiesterase type 4 family inhibitor. The recently obtained GEBR-32a crystal structure [10] evidenced that the long tail of the molecule (bearing the hydroxypropyl chain, with the chiral center and the morpholine end) extends toward the entrance of the enzyme active site. This feature may allow it to interact with the UCR2 or the CR3 regulatory domains that cap the catalytic pocket in the intact protein, thus possibly contributing to its inhibitory mechanism. To corroborate this hypothesis, we studied the relationship between the absolute configuration of GEBR-32a and its PDE4D inhibition. Therefore, we solved the two enantiomers via chiral HPLC and assigned their absolute configuration via ^1^H-NMR, using Mosher’s reagent, as chiral derivatizing agents. The enantiomers showed different potency against both the PDE4D catalytic domain and the long PDE4D3 isoform of the enzyme, providing evidence of the difference in the contacts that they may likely form with the regulatory domains of the enzyme.

Here, we show that the enantiomers of GEBR-32a interact with different potency with PDE4D and that (*S*)-GEBR 32a is the eutomer, in accordance with the crystallographic analysis. These results provide useful insights to guide improvements in binding potency and possible new avenues for the development of more potent and selective homochiral PDE4D inhibitors.

## Figures and Tables

**Table 1:** Analytical screening on Regispack IA (150 mm × 4.6 mm, 5 µm).

Mobile Phase ^a^ (*v*/*v*)	Flow	k_1_	k_2_	α	Rs
Hex/EtOH (90:10)	1 mL/min	3.05	3.07	N/A	N/A
Hex/EtOH (80:20)	1 mL/min	2.47	3.02	1.22	N/A
Hex/IPA (90:10)	1 mL/min	8.24	8.71	1.06	N/A
Hex/IPA (80:20)	1 mL/min	3.10	3.55	1.15	0.77
Hex/IPA (70:30)	1 mL/min	1.78	2.07	1.16	0.89
Hex/IPA (50:50)	1 mL/min	1.07	1.23	1.15	N/A
IPA	0.5 mL/min	0.87	1.56	1.79	2.61
IPA/EtOH (50:50)	0.5 mL/min	3.10	3.38	1.09	1.27
EtOH	0.5 mL/min	2.63	2.89	1.10	1.34
EtOH/ MeOH (50:50)	0.5 mL/min	2.24	2.42	1.08	1.09
MeOH	0.5 mL/min	0.48	0.53	1.10	N/A

^a^ 0.1% DEA added. k_1_: retention factor of the first eluted enantiomer, k_2_: retention factor of the second eluted enantiomer, α: resolution and Rs: factors.

**Table 2:** GEBR-32a enantiomers.

Cmpd	R_t_	*ee*	${\left[\alpha \right]}_{D}^{20}$ (*c* 0.2%, MeOH)	Isolated Amount	Yield
(–)-GEBR-32a	6.0 min	99.5%	−18.6	30 mg	43%
(+)-GEBR-32a	8.4 min	99.9%	+19.4	34 mg	48%

R_t_ = retention time and *ee* = enantiomeric excess.

**Table 3:** Nuclear magnetic resonance (NMR) analysis of derivatized and nonderivatized alcohols.

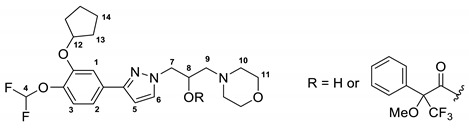			
**Proton** **(–)-GEBR-32a**	**1a**	**1b**	**Δδ = δ*_S_* − δ*_R_^a^***
H_5_	6.28	6.40	−0.12
H_6_	6.99	7.28	−0.29
H_7_	4.30–4.23	4.45–4.32	−0.15/0.09
			
H_9_	2.50	2.53	−0.03
H_10_	2.61–2.34	2.52–2.34	0.09
H_11_	3.57	3.60	−0.03
**Proton** **(+)-GEBR-32a**	**2a**	**2b**	**Δδ = δ*_S_* − δ*_R_***
H_5_	6.40	6.29	0.11
H_6_	7.36	6.99	0.37
H_7_	4.42–4.30	4.31–4.23	0.09/0.07
			
H_9_	2.48	2.51	−0.03
H_10_	2.51–2.34	2.62–2.36	−0.11/−0.02
H_11_	3.59	3.61	−0.02

*^a^* δ*_S_* = chemical shift of the (*S*)-methoxy-α-trifluoromethyl-α-phenylacetic acid (MTPA) ester and δ*_R_* = chemical shift of the (*R*)-MTPA ester.

**Table 4:** Inhibitory activity on both the catalytic domain and the full-length enzyme of racemic and enantiomers of GEBR-32a. PDE: phosphodiesterase.

	IC_50_ (µM) PDE4 cat	IC_50_ (µM) PDE4D3
(*R*)-(-)-GEBR-32a	31.8 ± 6.7	15.5 ± 2.7
(*S*)-(+)-GEBR-32a	19.5 ± 1.8	2.1 ± 0.3

## Supplementary Materials

The following are available online. Figure S1. Chromatographic profile at (λ = 254 nm) of racemic GEBR-32a in semi-preparative scale and chromatographic analysis of the pure enantiomers; Figure S2. Aromatic region of the NOESY spectrum of compound **1a**. The most important NOE contacts are evidenced; Figure S3. Aromatic region of the NOESY spectrum of compound **1b**. The most important NOE contacts are evidenced; Figure S4. Aromatic region of the NOESY spectrum of compound **2a**. The most important NOE contacts are evidenced; Figure S5. Aromatic region of the NOESY spectrum of compound **2b**. The most important NOE contacts are evidenced; Figure S6. The most intesting NOE contacts observed for compounds Mosher’s esters **2a** and **2b**; Table S1: NMR characterisation of non-derivatized GEBR-32a.

## References

[1] Prince M., Bryce R., Albanese E., Wimo A., Ribeiro W., Ferri C.P. (2013). The global prevalence of dementia: A systematic review and metaanalysis. Alzehimer’s Dement..

[2] Fillit H.M. (2000). The pharmacoeconomics of Alzheimer’s disease. Am. J. Manag. Care.

[3] Colucci L., Bosco M., Fasanaro A.M., Gaeta G.L., Ricci G., Amenta F. (2014). Alzheimer’s Disease Costs: What We Know and What We Should Take into Account. J. Alzheimer’s Dis..

[4] Jahn H. (2013). Memory loss in Alzheimer’s disease. Dialogues Clin. Neurosci..

[5] Kandel E.R. (2012). The molecular biology of memory: cAMP, PKA, CRE, CREB-1, CREB-2, and CPEB. Mol. Brain.

[6] Nabavi S.M., Talarek S., Listos J., Nabavi S.F., Devi K.P., de Oliveirad M.R., Tewari D., Argüelles S., Mehrzadi S., Hosseinzadeh A. (2019). Phosphodiesterase inhibitors say NO to Alzheimer’s disease. Food anche Chem. Toxicol..

[7] Ricciarelli R., Brullo C., Prickaerts J., Arancio O., Villa C., Rebosio C., Calcagno E., Balbi M., van Hagen B.T.J., Argyrousi E.K. (2017). Memory-enhancing effects of GEBR-32a, a new PDE4D inhibitor holding promise for the treatment of Alzheimer’s disease. Sci. Rep..

[8] Gulisano W., Tropea M.R., Arancio O., Palmeri A., Puzzo D. (2018). Sub-efficacious doses of phosphodiesterase 4 and 5 inhibitors improve memory in a mouse model of Alzheimer’s disease. Neuropharmacology.

[9] Blokland A., Heckman P., Vanmierlo T., Schreiber R., Paes D., Prickaerts J. (2019). Phosphodiesterase Type 4 Inhibition in CNS Diseases. Trends Pharmacol. Sci..

[10] Prosdocimi T., Mollica L., Donini S., Semrau M.S., Lucarelli A.P., Aiolfi E., Cavalli A., Storici P., Alfei S., Brullo C. (2018). Molecular Bases of PDE4D Inhibition by Memory-Enhancing GEBR Library Compounds. Biochemistry.

[11] Rossi D., Tarantino M., Rossino G., Rui M., Juza M., Collina S. (2017). Approaches for multi-gram scale isolation of enantiomers for drug discovery. Expert Opin. Drug Discov..

[12] Cosimelli B., Greco G., Laneri S., Novellino E., Sacchi A., Collina S., Rossi D., Cosconati S., Barresi E., Taliani S. (2018). Studies on enantioselectivity of chiral 4-acetylamino-6-alkyloxy-2-alkylthiopyrimidines acting as antagonists of the human A3 adenosine receptor. Medchemcomm.

[13] Rui M., Marra A., Pace V., Juza M., Rossi D., Collina S. (2016). Novel enantiopure sigma receptor modulators: Quick (semi-)preparative chiral resolution via hplc and absolute configuration assignment. Molecules.

[14] Rossi D., Marra A., Rui M., Brambilla S., Juza M., Collina S. (2016). “Fit-for-purpose” development of analytical and (semi)preparative enantioselective high performance liquid and supercritical fluid chromatography for the access to a novel σ 1 receptor agonist. J. Pharm. Biomed. Anal..

[15] Rossi D., Nasti R., Collina S., Mazzeo G., Ghidinelli S., Longhi G., Memo M., Abbate S. (2017). The role of chirality in a set of key intermediates of pharmaceutical interest, 3-aryl-substituted-γ-butyrolactones, evidenced by chiral HPLC separation and by chiroptical spectroscopies. J. Pharm. Biomed. Anal..

[16] Rossi D., Nasti R., Marra A., Meneghini S., Mazzeo G., Longhi G., Memo M., Cosimelli B., Greco G., Novellino E. (2016). Enantiomeric 4-Acylamino-6-alkyloxy-2 Alkylthiopyrimidines As Potential A3 Adenosine Receptor Antagonists: HPLC Chiral Resolution and Absolute Configuration Assignment by a Full Set of Chiroptical Spectroscopy. Chirality.

[17] Gaggeri R., Rossi D., Christodoulou M.S., Passarella D., Leoni F., Azzolina O., Collina S. (2012). Chiral Flavanones from Amygdalus lycioides Spach: Structural Elucidation and Identification of TNFalpha Inhibitors by Bioactivity-guided Fractionation. Molecules.

[18] Gaggeri R., Rossi D., Collina S., Mannucci B., Baierl M., Juza M. (2011). Quick development of an analytical enantioselective high performance liquid chromatography separation and preparative scale-up for the flavonoid naringenin. J. Chromatogr. A.

[19] Seco J.M., Quiñoá E., Riguera R. (2004). The Assignment of Absolute Configuration by NMR. Chem. Rev..

[20] Dale J.A., Mosher H.S. (1969). α-Methoxy-α-trifluoromethylphenylacetic acid, a versatile reagent for the determination of enantiomeric composition of alcohols and amines. J. Org. Chem..

[21] Fadia Y.S., Ashour M.L., Singa A.N.B., Wink M. (2019). A Comprehensive Review of Bioactive Peptides from Marine Fungi and Their Biological Significance. Mar. Drugs.

[22] Hub L., Mosher H.S. (1970). α-Methoxy-α-trifluoromethylphenylacetic acid. Configuration by asymmetric synthesis. J. Org. Chem..

[23] Sullivan G.R., Dale J.A., Mosher H.S. (1973). Correlation of configuration and fluorine-19 chemical shifts of α-methoxy-α-trifluoromethylphenyl acetate derivatives. J. Org. Chem..

[24] Peterson J.R., Bickford L.C., Morgan D., Kim A.S., Ouerfelli O., Kirschner M.W., Rosen M.K. (2004). Chemical Inhibition of N-WASP by stablization of a native autoinhibited conformation. Nat. Struct. Mol. Biol..

[25] Vasile F., Civera M., Belvisi L., Potenza D., Tiana G. (2016). Thermodynamically–weighted conformational ensemble of cyclic RGD peptidomimetics from NOE data. J. Phys. Chem. B.

[26] Vasile F., Panigada M., Siccardi A., Potenza D., Tiana G. (2018). A Combined NMR-Computational Study of the Interaction between Influenza Virus Hemagglutinin and Sialic Derivatives from Human and Avian Receptors on the Surface of Transfected Cells. Int. J. Mol. Sci..

[27] Burgin A.B., Magnusson O.T., Singh J., Witte P., Staker B.L., Bjornsson J.M., Thorsteinsdottir M., Hrafnsdottir S., Hagen T., Kiselyov A.S. (2010). Design of phosphodiesterase 4D (PDE4D) allosteric modulators for enhancing cognition with improved safety. Nat. Biotechnol..

[28] Bieniossek C., Imasaki T., Takagi Y., Berger I. (2012). MultiBac: Expanding the research toolbox for multiprotein complexes. Trends Biochem. Sci..

[29] Chock S.P., Huang C.Y. (1984). An optimized continuous assay for cAMP phosphodiesterase and calmodulin. Anal. Biochem..

