# Supplementary material for: Insight into GEBR-32a: Chiral Resolution, Absolute Configuration and Enantiopreference in PDE4D Inhibition
Source: Molecules. 2020 Feb 19;25(4):935. doi: 10.3390/molecules25040935 (PMC7070305; doi:10.3390/molecules25040935)
Supplement: Supplementary file 1 [file molecules-25-00935-s001.pdf]

Supplementary material

S1. Synthesis of GEBR-32a

Synthesis of racemic GEBR-32a is reported in scheme S1.

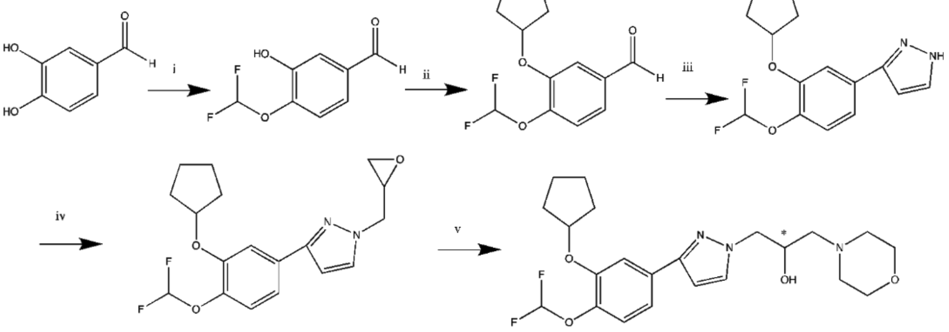

Scheme SI: synthesis of racemic GEBR-32a. Reagent and conditions: (i) $CClF_2COOCH_3$, Cs_2CO_3, anydrous DMF, 300 W, 90 °C, 25 min; (ii) bromocyclopentane, K_2CO_3/KI, an. DMF, 65 °C, 22 h; (iii) p-toluenesulfonyl hydrazide, anhydrous acetonitrile, RT, 1 h; 5N NaOH solution RT, 20 min.; 1-vinylimidazole, 50 °C, 48 h, yield= 45%; (iv) epichlorohydrin 0–5 °C; then, TEA, stirred until 25 °C, then 70 °C, 6 h, yield= 58%; (v) morpholine excess, 50–60 °C, 18 h, yield= 56%.

S2. Scale up of the chromatographic conditions to semipreparative scale

The analytical method set up during this work was properly scaled up to allow the separation of the different fraction. In detail, Regispack IA (1 cm × 25 cm, 5 μm) was exploited as column, at a flow rate of 2 mL/min, with an injection volume of 1mL (concentration of the injected solution: 10mg/mL).

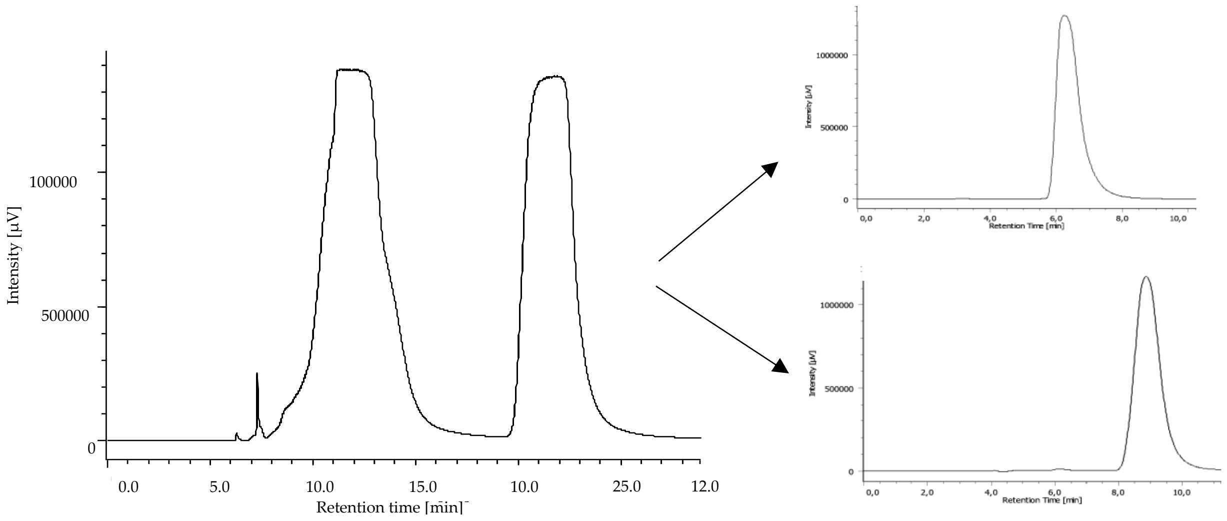

Figure S1. Chromatographic profile at (λ=254nm) of racemic GEBR-32a in semi-preparative scale and chromatographic analysis of the pure enantiomers.

S3. NMR characterizarion of GEBR- 32a

^{1}H-NMR and ^{13}C-NMR signals of non-derivatized GEBR-32a are reported in table S1.
The more interesting regions (with the more diagnostic contacts) of NOE bidimensional spectra are reported in figure S2-S5.

Table S1: NMR characterisation of non-derivatized GEBR-32a

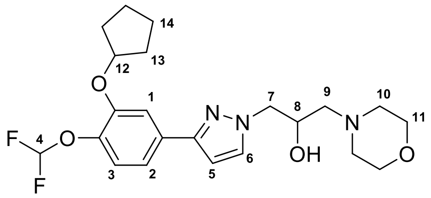

Atom	^{1}H (ppm)	^{13}C (ppm)
**1**	7.34 (d, J= 1.56 Hz)	112.6
**2**	7.2 (dd, J= 8.2, 1.56 Hz)	118.1
**3**	7.08 (d, J= 8.2 Hz)	123.1
**4**	6.47(d, $J_{H\text{-}F}$= 75.9 Hz)	115.5
**5**	6.44 (d, J= 2.26 Hz)	102.5
**6**	7.45 (d, J= 2.26 Hz)	131.9
**7**	4.22 - 4.1 (m)	55.7
**8**	4.1 (m)	66.1
**9**	2.35 (m)	61.5
**10**	2.56 – 2.41 (m)	54.1
**11**	3.65 (m)	66.7
**12**	4.84 (m)	80.6
**13**	1.86 (m)	32.6
**14**	1.76 – 1.58 (m)	23.9

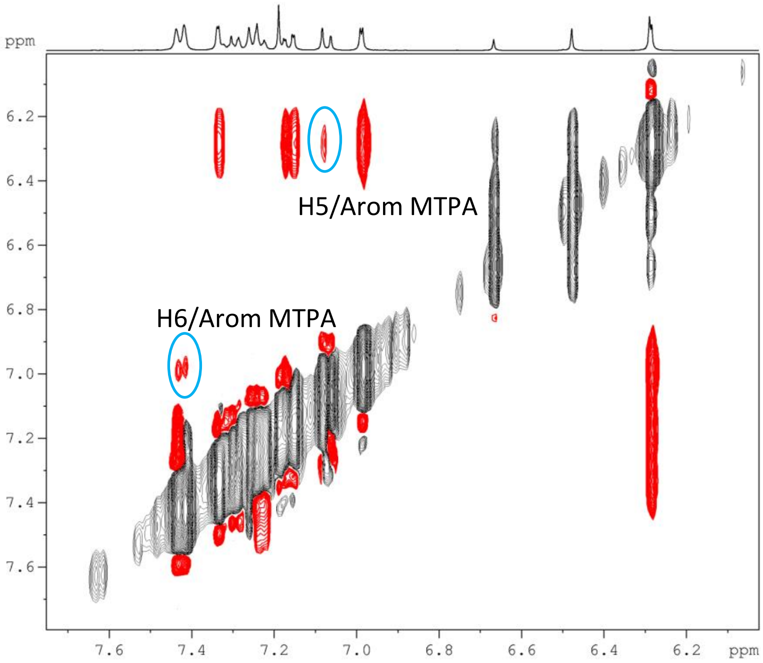

Figure S2. Aromatic region of the NOESY spectrum of compound **1a**. The most important NOE contacts are evidenced.

Figure S3. Aromatic regio [illegible] NOE contacts are

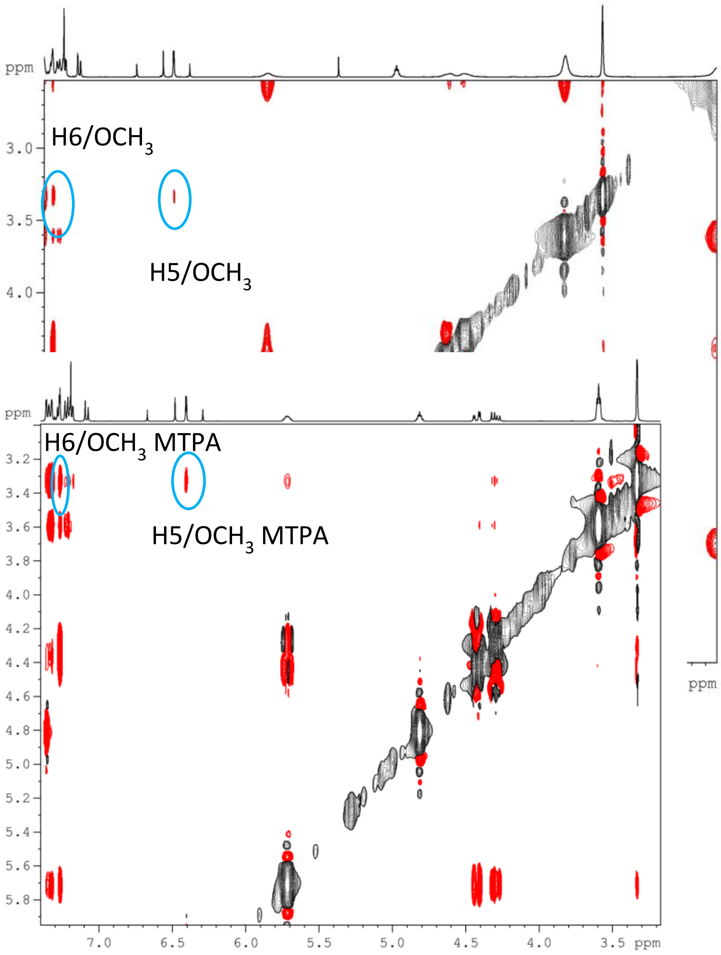

Figure S4. Aromatic region of the NOESY spectrum of compound **2a**. The most important NOE contacts are evidenced.

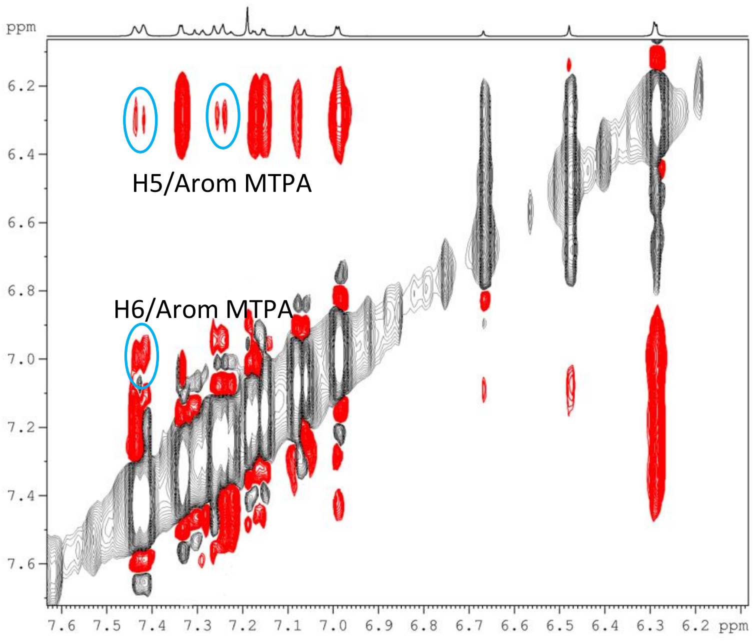

Figure S5. Aromatic region of the NOESY spectrum of compound **2b**. The most important NOE contacts are evidenced.

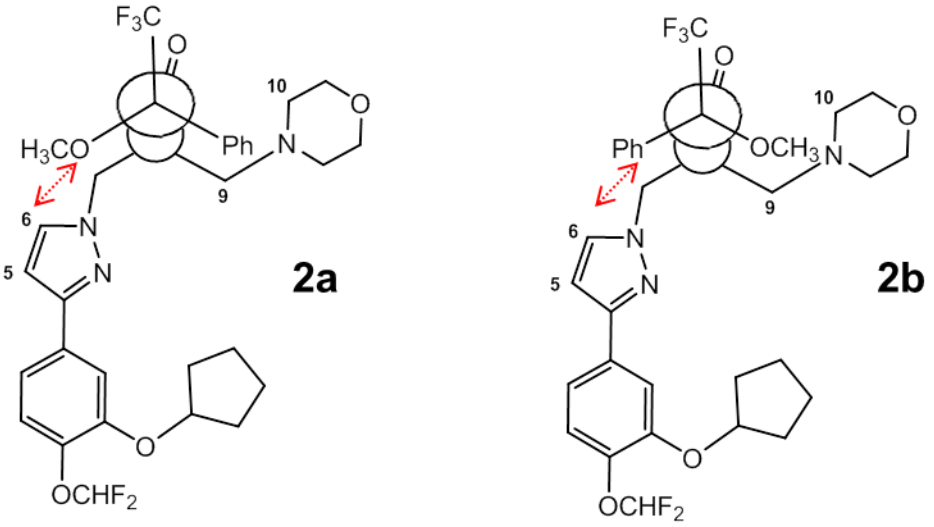

Figure S6. The most intesting NOE contacts observed for compounds Mosher's esters **2a** and **2b.**
